# A Biodegradable Zinc Alloy Membrane with Regulation of Macrophage Polarization for Early Vascularized Bone Regeneration

**DOI:** 10.34133/bmr.0223

**Published:** 2025-07-02

**Authors:** Li Yi, Ruimin Tang, Chunsheng Shao, Chang Chen, Jiangjie Tang, Luman Liao, Liangjian Chen

**Affiliations:** ^1^ Department of Stomatology, The Third Xiangya Hospital, Central South University, Changsha, Hunan 410013, People’s Republic of China.; ^2^ Department of Endodontics, Changsha Stomatological Hospital, Changsha 410004, Hunan Province, People’s Republic of China.

## Abstract

Guided bone regeneration (GBR) has become a standard modality for treating localized jawbone defects in the clinic. For optimal bone regeneration, the GBR membrane must be biodegradable and exhibit superior mechanical properties. Zinc, a biodegradable metal, has demonstrated marked potential for use in GBR membranes. To address the insufficient mechanical properties of pure zinc membranes, a Zn-0.3Fe-0.05Mg membrane with enhanced mechanical performance was developed in this study. The Young’s modulus, hardness, ultimate tensile strength, and elongation at break of the Zn-0.3Fe-0.05Mg membrane were 47.94 ± 7.38 GPa, 0.58 ± 0.08 GPa, 294.07 ± 7.16 MPa, and 20.67% ± 0.15%, respectively, all of which were superior to those of the pure zinc membrane. Moreover, at a concentration of less than 25%, the membrane extract was not cytotoxic, while in the concentration range of 10% to 25% (zinc concentration of 37.33 ± 3.50 to 93.33 ± 8.75 μM), the membrane extract induced the M2 polarization of Raw264.7 cells. Then, at membrane extract concentrations of 10% to 25%, the osteogenic differentiation of MC3T3-E1 cells and vascularization of human umbilical vein endothelial cells (HUVECs) were promoted in the Raw264.7–MC3T3-E1 and Raw264.7–HUVEC coculture systems. Furthermore, scanning electron microscopy, microcomputed tomography, and histological analyses revealed that the Zn-0.3Fe-0.05Mg membrane promoted M2 macrophage polarization and angiogenesis in vivo, thereby facilitating early bone formation after 2 to 4 weeks. These findings suggest that the Zn-0.3Fe-0.05Mg membrane can degrade and release Zn^2+^ to regulate M2 macrophage polarization and promote early vascularized bone regeneration, showing the potential of Zn-0.3Fe-0.05Mg membranes as ideal GBR membranes.

## Introduction

Guided bone regeneration (GBR) is a surgical technique that isolates soft tissues through physical barriers and promotes bone regeneration. It is primarily used to repair alveolar bone defects or areas with insufficient bone volume, creating adequate bone volume conditions for subsequent dental implant placement. GBR membranes are essential surgical implant materials that can prevent the migration of connective tissue and epithelial cells to the defect and provide space and time for bone regeneration [1].

The GBR membranes commonly used in clinical practice at present can be classified as degradable membranes or nondegradable membranes. Collagen membranes are commonly used degradable membranes with good biocompatibility and degradability [2]. However, collagen membranes are rapidly degraded and absorbed under the effects of collagenase, bacterial proteases, and macrophage-derived enzymes [2,3], which makes achieving long-term barrier and space maintenance functions difficult. Nonabsorbable membranes, such as titanium and polytetrafluoroethylene, have high stiffness and carry exposure risks after implantation. Additionally, they need to be removed by a second operation, which remarkably increases patient pain, infection risk, and medical costs. An ideal barrier membrane should have appropriate mechanical strength, good biocompatibility, and biodegradability [1]. However, the GBR membranes currently in use do not meet these requirements.

Zinc (Zn) has been identified as a new degradable material for GBR in recent years, with a moderate degradation rate and good biocompatibility [4]. However, Guo et al.’s animal experiments revealed that some pure zinc membranes collapse due to insufficient mechanical properties [5,6]. Therefore, alloying is essential for improving the mechanical properties of pure zinc membranes. Ferrum (Fe) and magnesium (Mg) are also biodegradable metals with good biocompatibility [7], which makes them candidate Zn alloying elements. Su et al. [8] reported that a Zn-0.4Fe alloy displayed remarkably improved mechanical properties and promoted uniform degradation, but its degradation was slower than that of pure zinc. Shi et al. [9] prepared a Zn-0.3Fe alloy with excellent mechanical properties, corrosion resistance, and biocompatibility. However, the degradation properties of Zn–Fe need to be further improved. The potentials of the Mg_2_Zn_11_ and MgZn_2_ phases are lower than that of the Zn matrix. Therefore, the second phase is preferentially corroded as the anode, leading to accelerated degradation [10]. Jin et al. reported that trace amounts of magnesium (<1 wt%) can increase the degradation rate of Zn–Mg alloys over time after implantation. This facilitates sufficient and rapid degradation of the implanted material once its usage requirements have been met [11]. Wang et al. [12] noted that upon optimization of the magnesium content in Zn–Mg alloys, Zn-0.05Mg not only exhibited better mechanical properties but also displayed good biocompatibility and antibacterial activity than pure zinc. Therefore, using a Zn–Fe–Mg alloy may be an effective strategy to obtain an alloy with good mechanical properties and a suitable degradation rate.

An important research direction in the biological activity of implant materials is the regulation of macrophage polarization. Depending on the stimulus, activated macrophages can polarize into distinct phenotypes and release a range of inflammatory and chemotactic factors, which play crucial roles in the inflammatory response and tissue repair processes shortly after material implantation [13]. Further studies have shown that M2 macrophages play important regulatory roles in the early stage of vascularized bone regeneration [14]. M2 macrophages can promote the osteogenic differentiation of mesenchymal stem cells by secreting IL-10 and TGF-β1 [15]. Zhao et al. [16] reported that M2-type Raw264.7 cells promote the osteogenic differentiation of MC3T3-E1 cells by secreting oncostatin M and bone morphogenetic protein 2 (BMP-2). In addition, macrophages play key roles in neovasculature formation and maturation [17]. Graney et al. reported that M1 macrophages promote blood vessel formation during the early stage (1 d) and cause blood vessel degeneration during the middle and late stages (3 d). M2 macrophages play important roles in the maturation and stability of blood vessels [18].

Recent studies have shown that zinc ions have immunomodulatory effects. Zinc-containing biomaterials, such as zinc alloy materials, zinc-based ceramics, and zinc-based metal–organic frameworks, can promote bone defect repair by regulating the immune response [19]. Qian et al. [20] prepared a zinc-containing hydrogel to induce M2 macrophage polarization. Liu et al. [21] added zinc ions to the surface of sulfated polyetheretherketone biomaterials to promote the polarization of macrophages to the M2 phenotype, which is conducive to the osteogenic differentiation of bone marrow mesenchymal stem cells. However, the effects of Zn-0.3Fe-0.05Mg alloy membranes on macrophage polarization are still unclear, and the mechanisms by which they promote early vascularized bone regeneration need to be further explored.

**Fig. 1.**
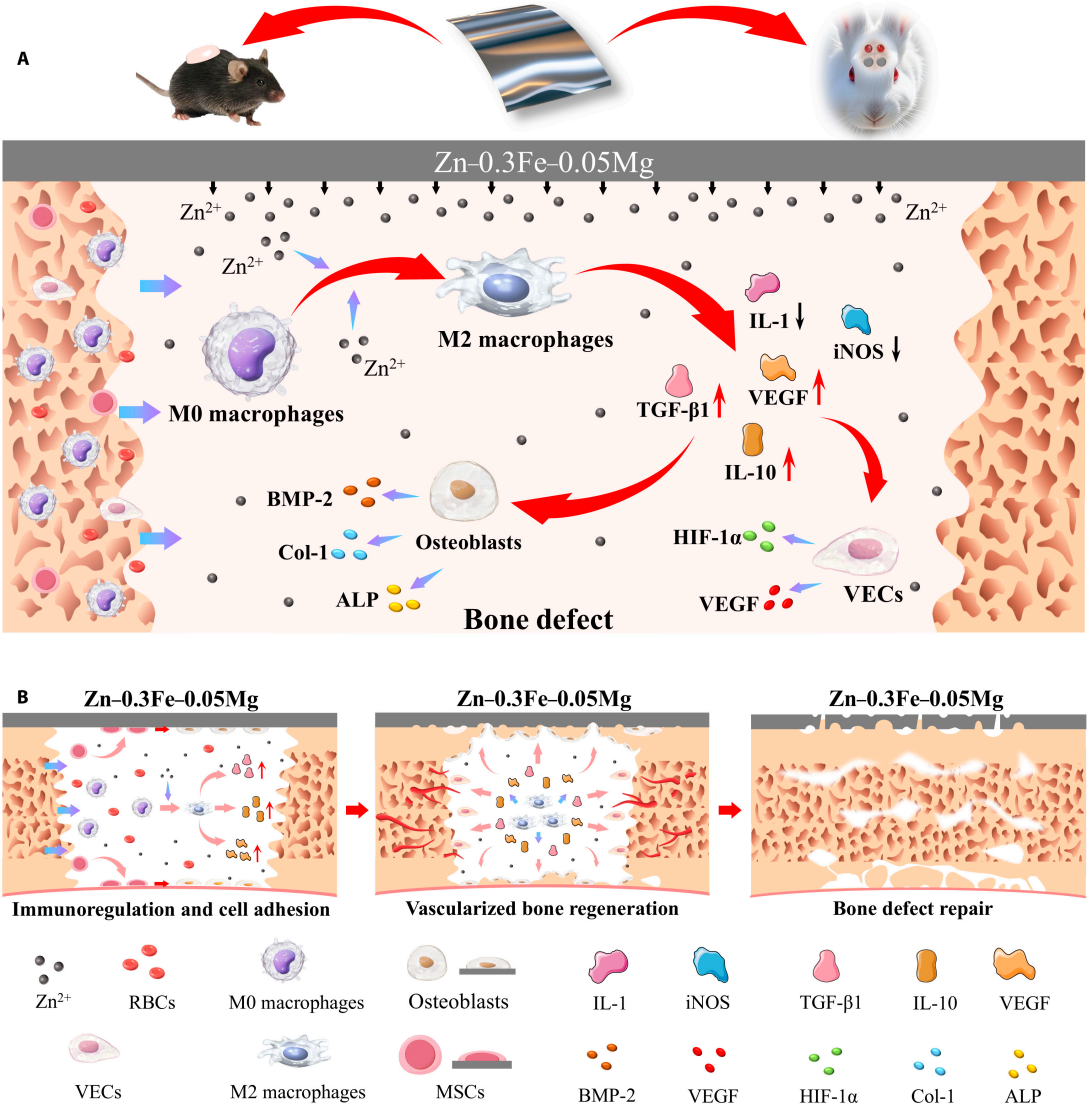
Schematic diagram illustrating the mechanism by which Zn-0.3Fe-0.05Mg membranes influence the osteoimmunomodulation cascade during the repair of bone defects. (A) Zinc membranes promote osteoblast differentiation and endothelial vascularization by mediating macrophage polarization through the slow release of zinc ions. (B) The process by which the zinc alloy membrane promotes the repair of critical bone defects. BMP-2, bone morphogenetic protein 2; Col-1, collagen-1; ALP, alkaline phosphatase; iNOS, inducible nitric oxide synthase; VEGF, vascular endothelial growth factor; HIF-1α, hypoxia-inducible factor-1 alpha; VECs, vascular endothelial cells; RBCs, red blood cells; MSCs, mesenchymal stem cells.

Therefore, to develop an ideal GBR membrane with excellent mechanical properties and a suitable degradation rate, this work developed a novel biodegradable Zn-0.3Fe-0.05Mg membrane. Firstly, the differences in microstructure and mechanical properties between the Zn-0.3Fe-0.05Mg and Zn membranes were compared. Then, the effects of the Zn-0.3Fe-0.05Mg membrane extract on the osteoimmunomodulation and degradation behavior in vivo and in vitro were investigated. Finally, the mechanism by which the Zn-0.3Fe-0.05Mg membrane regulates macrophage polarization to promote early vascularized bone regeneration was clarified (Fig. 1). Thus, the biodegradable Zn-0.3Fe-0.05Mg membrane with mechanical properties and immunoregulatory functions could be a potential candidate for GBR.

## Materials and Methods

### Material preparation

Zinc powder, magnesium powder, and reduced iron powder, all with purities ≥99.95%, were ball milled and mixed for 6 h with a nominal composition of Zn-0.3Fe-0.05Mg (wt%). The blended powder was subsequently pressed, molded, and solid-phase sintered. The sintering temperature was 400 °C, the sintering time was 24 h, the sintering pressure was 40 MPa, the heating rate was 50 °C/min, and the cooling rate was 10 °C/min. The alloy composition was 99.63 ± 0.047 wt% Zn, 0.28 ± 0.024 wt% Fe, and 0.048 ± 0.0029 wt% Mg, detected by inductively coupled plasma–optical emission spectrometry (ICP–OES; SPECTRO BLUE, Germany). The obtained ingots were homogenized at 350 ± 20 °C and then extruded into zinc alloy sheets with a thickness of 5 mm at 300 ± 20 °C with an extrusion ratio of 5:1. After the sheets were held at 200 ± 20 °C for 30 min, 60-μm-thick Zn-0.3Fe-0.05Mg membranes were obtained after multiple rolling processes, controlling the shape variation in each pass at 20% to 30%. For subsequent experiments, the alloy membranes were subjected to grinding and polishing treatments to control the Ra value within 1.6 to 2.

### Characterization of the microstructure and mechanical tests

The microstructure and composition of the membranes were further evaluated using field-emission scanning electron microscopy (SEM; Zeiss, Sigma 300, Germany) coupled with energy-dispersive spectroscopy.

Nanoindentation tests were conducted with NanoTest Vantage (Micro Materials, UK). The applied maximum load, drift rate, and peak holding time were set to 20 mN, 1.2 nm/s, and 120 s, respectively. Three samples’ stress–strain curves per group were analyzed to assess the hardness and elasticity modulus.

The tensile tests were conducted on samples 4 mm in width and 28 mm in length using a universal testing machine (Instron 5982, USA) with a displacement rate of 1 mm/min at room temperature.

### Immersion test

The Zn-0.3Fe-0.05Mg membranes were immersed in simulated body fluid (SBF; G-clone, China) at 37 °C. According to the ASTM G31-72 standard, the ratio of the soaking solution volume to the surface area was 20 ml/cm^2^ [22].

Furthermore, the cumulative release of zinc, magnesium, and iron ions was determined after 0.5, 1, 3, 5, 7, 14, 21, and 28 d of treatment in SBF via inductively coupled plasma–optical emission spectrometry (ICP–OES; SPECTRO BLUE, Germany), and the pH values were measured with a pH meter (Guangzhou Deweijia Biotechnology, China).

Moreover, the corrosion morphologies of the Zn-0.3Fe-0.05Mg membranes were observed after 3, 7, 14, and 28 d of immersion by SEM (Zeiss Sigma 300, Germany). At each time point, the corrosion products were removed and ultrasonically cleaned in a solution of chromic acid (200 g/l CrO_3_; Macklin, China) at 37 °C for 5 min, after which the corrosion rates were estimated based on weight loss.

### In vitro cell experiments

#### Preparation of the extract

The Dulbecco’s modified Eagle medium (DMEM; Gibco, USA) used in this study contained 10% fetal bovine serum (Gibco, USA) and 1% penicillin–streptomycin (Gibco, USA). After low-temperature plasma sterilization (LK/MJQ-100, China), the Zn-0.3Fe-0.05Mg membranes were immersed in fresh culture medium at a density of 1.25 cm^2^/ml in an incubator at 37 °C for 72 h to obtain the extract [6]. The zinc, magnesium, and iron ions in the membrane extract and fresh complete medium were measured via ICP–OES, and the pH values were measured with a pH meter (Guangzhou Deweijia Biotechnology, China).

#### Macrophage cytotoxicity assessment

Raw264.7 cells (Cell Bank of the Chinese Academy of Sciences) were cultured in 96-well plates at 3,000 cells/well density. After overnight culture, the DMEM was replaced with 100%, 50%, 25%, or 10% extract, which was replaced daily. Following 2 or 4 d of culture, the cells were incubated in 10% Cell Counting Kit-8 reagent without serum for 2 h. Afterward, an enzymatic marker was used to assess the cell viability by measuring the absorbance at 450 nm.

Raw264.7 cells were seeded into 24-well plates (10^5^ cells/well). The DMEM was replaced with 100%, 50%, 25%, or 10% extract for 4 d after overnight culture. Then, the cells were stained with live/dead staining reagent (Beyotime, China). A fluorescence microscope was used to capture images of the cells.

#### Effect of the extract on macrophage polarization

Based on the results of the cytotoxicity experiments, 25%, 20%, 15%, 10%, and 5% membrane extracts were selected for further study. Real-time quantitative polymerase chain reaction (RT-qPCR), immunofluorescence (IF), and flow cytometry (FCM) were conducted to evaluate macrophage polarization.

Briefly, Raw264.7 cells were incubated with 25%, 20%, 15%, 10%, or 5% membrane extract in a 6-well plate (10^5^ cells/well) for 3 d. Then, an RNAfast200 kit (Fastagen, China) was used for RNA extraction following the manufacturer’s instructions. A NanoDrop 2000 spectrophotometer (Thermo Scientific, USA) was subsequently used to determine the total RNA concentration.

Moreover, SYBR Green PCR MasterMix (Vazyme, China) was used to measure the gene expression of M1 (inducible nitric oxide synthase [iNOS] and IL-1) and M2 (IL-10, CD206, vascular endothelial growth factor [VEGF], and TGF-β1) macrophage markers. The sequences of the primers used are shown in Table S1. Glyceraldehyde-3-phosphate dehydrogenase was used as an internal control, and the Ct method was used to determine the fold change.

Raw264.7 cells were incubated with 25%, 20%, 15%, 10%, or 5% membrane extract in a 6-well plate (10^5^ cells/well) for 3 d. Then, the cells were fixed with 4% paraformaldehyde (Servicebio, China) for 15 min and permeabilized with 0.5% Triton X-100 (Beyotime, China). After blocking with 1% bovine serum albumin (BSA; Beyotime, China) for 15 min, the cells were incubated with primary antibodies against CD206 (Proteintech Group, 1:200, China) and iNOS (Proteintech Group, 1:200, China) at 4 °C overnight, followed by incubation with goat anti-rabbit Alexa Fluor 488 (Abcam, 1:500, China) and goat anti-mouse Alexa Fluor 594 (Abcam, 1:500, China) secondary antibodies and further incubation at ambient temperature for 2 h. 4′,6-Diamidino-2-phenylindole (DAPI) solution (Biosharp, China) was subsequently used to stain the nuclei for 10 min, and a fluorescence microscope (Carl Zeiss AG, Germany) was used to observe the morphology of the Raw264.7 cells.

Furthermore, Raw264.7 cells were fixed and permeabilized using Fix & Perm Kit (Liankebio, China). After incubation with antibodies against CD206 (eBioscience, 1:100, USA) and iNOS (eBioscience, 1:200, USA), the cells were examined by FCM (Cytek NL3000, Cytek Biosciences, USA), and the percentages of M1 and M2 macrophages were determined using FlowJo 10.8.1 (BD Biosciences, USA).

#### Effect of the extract on MC3T3-E1 cells

MC3T3-E1 cells (Cell Bank of the Chinese Academy of Sciences) were incubated with 25%, 20%, 15%, 10%, or 5% membrane extract in a 6-well plate (10^5^ cells/well) for 3 d. The expression of osteogenesis-related genes, such as alkaline phosphatase (ALP), BMP-2, collagen-1 (Col-1), and runt-related transcription factor 2 (RUNX2), was measured as described in the “Effect of the extract on macrophage polarization” section. The sequences of the primers used are shown in Table S1.

#### Effect of the extract on HUVECs

Human umbilical vein endothelial cells (HUVECs; Cell Bank of the Chinese Academy of Sciences) were incubated with 25%, 20%, 15%, 10%, or 5% membrane extract in a 6-well plate (10^5^ cells/well) for 3 d. The expression of angiogenesis-related genes, such as VEGF and hypoxia-inducible factor-1 alpha (HIF-1α), was measured as described in the “Effect of the extract on macrophage polarization” section. The sequences of the primers used are shown in Table S1.

#### Construction of the cell coculture systems

To evaluate the effects of Raw264.7 cell polarization on MC3T3-E1 cells and HUVECs, 0.4-μm transwell chambers (Corning, USA) were used to construct Raw264.7–MC3T3-E1 cell and Raw264.7–HUVEC coculture systems. Raw264.7 cells and MC3T3-E1 cells or HUVECs were seeded above and below the transwell chambers, respectively (10^5^ cells/well).

#### Osteogenic activity of MC3T3-E1 cells in the coculture system

Three days after adding 25%, 20%, 15%, 10%, or 5% membrane extract to the coculture system, RT-qPCR was employed to examine the expression of osteogenesis-related genes in the cocultured MC3T3-E1 cells. Methods similar to those described in the “Effect of the extract on macrophage polarization” section were used to detect the expression of genes such as BMP-2, Col-1, and RUNX2. The sequences of the primers used are shown in Table S1.

Three days after the addition of 25%, 20%, 15%, 10%, or 5% membrane extract, the cocultured MC3T3-E1 cells were fixed with 4% paraformaldehyde (Servicebio, China) for 15 min and permeabilized with 0.5% Triton X-100 (Beyotime, China). After blocking with 1% BSA (Beyotime, China) for 15 min, the cells were incubated with a primary antibody for osteopontin (OPN; 1:200; Proteintech Group) at 4 °C overnight, followed by the addition of the secondary antibody goat anti-rabbit Alexa Fluor 488 (Abcam, 1:500; China) and further incubation at ambient temperature for 2 h. DAPI solution (Biosharp, China) was subsequently used to stain the nuclei for 10 min, and a fluorescence microscope (Carl Zeiss AG, Germany) was used to observe the morphology of the MC3T3-E1 cells. The control group included cocultured MC3T3-E1 cells with standard medium under the same conditions. The mean fluorescence intensity of OPN was measured with ImageJ 1.8.0 (National Institutes of Health, USA).

Three days after the addition of 25%, 20%, 15%, 10%, or 5% membrane extract, the ALP activity MC3T3-E1 cells (10^5^ cells/well) in the coculture system was measured using a BCIP/NBT ALP color development kit (Beyotime, China) and an ALP assay kit (Beyotime, China) according to the manufacturer’s instructions.

#### Vascularization of HUVECs in the coculture system

Three days after adding 25%, 20%, 15%, 10%, or 5% membrane extract, RT-qPCR was used to measure the expression of angiogenesis-related factors in HUVECs, such as VEGF and HIF-1α, in the coculture system, using methods similar to those described in the “Effect of the extract on macrophage polarization” section. The sequences of the primers used are shown in Table S1.

Three days after the addition of 25%, 20%, 15%, 10%, or 5% membrane extract, the VEGF expression in the HUVECs in the coculture system was evaluated via IF, as described in the “Osteogenic activity of MC3T3-E1 cells in the coculture system” section. The mean fluorescence intensity of VEGF was measured with ImageJ 1.8.0 (National Institutes of Health, USA).

Macrophage-conditioned medium (CM) was prepared by incubating Raw264.7 cells with 25%, 20%, 15%, 10%, or 5% membrane extract for 3 d, and then the supernatants were collected. HUVECs were seeded in Matrigel-coated (Corning, USA) 96-well plates (2 × 10^4^ cells/well) in macrophage CM for 4 h, after which the HUVECs were stained with calcein AM for 15 min. A fluorescence microscope was used to observe the morphology of the HUVECs, and the total number of junctions and total vessel length were evaluated with Angio Tool 0.6a (National Cancer Institute, USA).

### In vivo animal experiments

#### Mouse air pouch model

These animal experiments were approved by the Animal Research Committee of Central South University (animal ethics approval number: CSU-2022-0590). Six mice (C57BL/6, male, 8 weeks old) were evenly divided into 2 groups, and the in vivo immunomodulatory effects of the Zn-0.3Fe-0.05Mg membranes were investigated after establishing an air pouch model. Three mice from each group were subjected to IF staining. The mice were anesthetized with 3% pentobarbital (1 ml/kg) via intraperitoneal injection, and 5 ml of sterile air was injected into the dorsal area. Another 3 ml of sterile air was injected 5 d later. One day after the second injection, a surgical incision was made in the middle of the pouch to implant the zinc alloy membrane. Mice with an air pouch that did not receive an implanted membrane were used as controls. All surgical procedures were performed aseptically, and the mice were sacrificed 3 d after surgery.

For in vivo IF staining, the skin tissue containing the pouch was harvested, and the Zn-0.3Fe-0.05Mg membranes were carefully removed. The tissue was subsequently fixed with 4% paraformaldehyde for 0.5 h, and nonspecific antigen binding was blocked with 1% BSA. To determine the expression levels of M0 (CD68; 1:200, Proteintech Group, China), M1 (iNOS; 1:200, Proteintech Group, China), and M2 (CD206; 1:200, CST, USA) macrophage markers, primary and secondary antibodies were utilized according to the same procedures described in the “Effect of the extract on macrophage polarization” section. IF images were obtained via fluorescence microscopy.

#### New Zealand rabbit skull defect model

These animal experiments were approved by the Animal Research Committee of Central South University (animal ethics approval number: CSU-2023-0279). New Zealand rabbits (2.5 to 3 kg, male) were used to evaluate the extent of bone regeneration induced by the Zn-0.3Fe-0.05Mg membranes. The rabbits from each group were utilized for the skull bone defect model, and all of the samples obtained were subjected to microcomputed tomography (micro-CT) and histological analyses. The rabbits were anesthetized with 3% pentobarbital (1 ml/kg) via intravenous injection. Prior to surgery, the hair was shaved, and the area was disinfected. A manual drill (diameter of 8 mm) was used to drill through the skull, and sterilized Zn-0.3Fe-0.05Mg and collagen membranes were implanted. After suturing, ceftriaxone sodium was injected intramuscularly. Rabbits were sacrificed 4 d and 1, 2, 4, and 12 weeks after surgery. Rabbits subjected to bone defect establishment but not membrane implantation were used as blank controls.

The membranes obtained 4 d and 12 weeks after surgery were fixed with 10% buffered formalin and observed via SEM to assess cell adhesion.

The soft tissue near the defect area and major organs (brain, lung, liver, spleen, heart, and kidney) were collected 12 weeks after surgery for hematoxylin and eosin (HE) staining to evaluate the biocompatibility of the Zn-0.3Fe-0.05Mg membrane in vivo.

Soft tissue samples near the membrane taken 1 week after surgery were processed for immunohistochemical staining to investigate pathological features and vascularization.

For the micro-CT analysis, the regions of interest were full-thickness cylinders 8 mm in diameter, which were selected based on the dimensions of the bone defect sites. The skulls that were implanted with the Zn-0.3Fe-0.05Mg membranes were analyzed by micro-CT (Kaisheng, China). The bone volume fraction (bone volume/total volume [BV/TV]) and membrane volume were analyzed with ZZKS-MicroCT4.1 (Kaisheng, China) 2, 4, and 12 weeks after surgery. The corrosion rates were estimated based on volume loss. Then, skull tissues implanted with Zn-0.3Fe-0.05Mg membranes were made into stiff tissue sections and stained with Goldner’s trichrome.

### Statistical analysis

GraphPad Prism 8.0 (GraphPad Software, USA) was used for statistical analysis. All data are expressed as mean ± standard deviation. *t* tests were used for comparisons between 2 groups. Comparisons among multiple groups were performed using one-way analysis of variance, and differences were considered statistically significant at *P* < 0.05.

## Results

### Microstructural characterization and mechanical properties

**Fig. 2.**
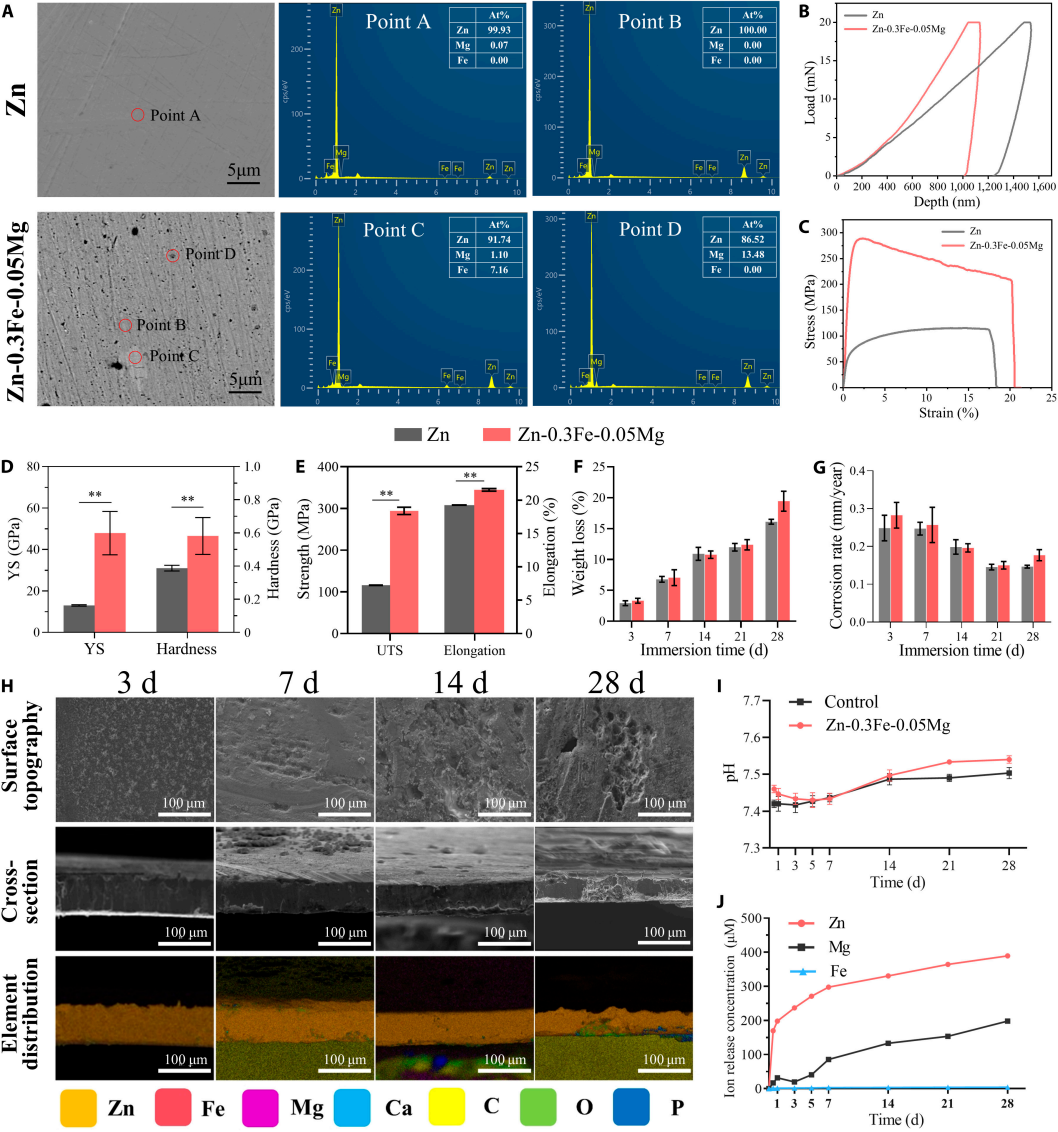
Microstructure, mechanical properties, and degradation behavior of the Zn-0.3Fe-0.05Mg membrane. (A) Microstructure and energy-dispersive spectroscopy (EDS) results of the pure Zn and Zn-0.3Fe-0.05Mg membranes in backscattering mode (points A to D are marked with red circles). (B and C) Representative load–depth and stress–strain curves of the pure Zn and Zn-0.3Fe-0.05Mg membranes. (D and E) Young’s modulus (YS; GPa), hardness (GPa), ultimate tensile strength (UTS; MPa), and elongation (%) at break of the pure Zn and Zn-0.3Fe-0.05Mg membranes. (F and G) Weight loss (%) and corrosion rate (mm/year) of the pure Zn and Zn-0.3Fe-0.05Mg membranes. (H) The corrosion morphology of the Zn-0.3Fe-0.05Mg membrane after soaking in simulated body fluid (SBF). (I and J) pH and released ion content in the membrane extract. The results are shown as mean ± SD. Comparisons were made via one-way analysis of variance (ANOVA) (*P < 0.05; **P < 0.01).

SEM images of the microstructures of the pure Zn and Zn-0.3Fe-0.05Mg membranes are shown in Fig. 2A. The pure zinc membrane had a uniform composition. In contrast, the Zn-0.3Fe-0.05Mg membrane had 2 secondary phases, marked with C and D, respectively. Energy-dispersive spectroscopy analysis revealed that the secondary phase labeled C, with a diameter of approximately 5 μm, may be FeZn_13_ [23]; the secondary phase labeled B, with a diameter of less than 1 μm, may be Mg_2_Zn_11_ [24].

Figure 2B and C show the load–depth and stress–strain curves of the pure Zn and Zn-0.3Fe-0.05Mg membranes, respectively. As shown in Fig. 2D, the Young’s modulus (YS) and hardness of the Zn membrane were 13.03 ± 0.23 and 0.39 ± 0.01 GPa, respectively. After alloying, the YS and hardness of the Zn-0.3Fe-0.05Mg membrane increased significantly, up to 47.94 ± 7.38 and 0.58 ± 0.08 GPa, respectively (Fig. 2E). Moreover, the tensile test results show that the ultimate tensile strength and elongation of the Zn-0.3Fe-0.05Mg membrane were 294.07 ± 7.16 MPa and 20.67% ± 0.15%, respectively, significantly greater than those of the pure Zn membrane. The results showed that alloying improved the mechanical properties of the Zn-0.3Fe-0.05Mg membrane and benefited the maintenance of the morphology of the GBR membrane after its implantation in vivo.

### Degradation behavior of the membranes in vitro

The corrosion morphology of the Zn-0.3Fe-0.05Mg membrane after immersion in SBF is shown in Fig. 2H. During the initial 3 d of immersion, intricate formations resembling needle clusters emerged on the surface of the membrane, and no corrosion spots were visible. Over the subsequent 21 d, the initially scattered corrosion spots transformed into extensively corroded sheets characterized by steady growth in the size and depth of the affected area.

Cross-sectional analysis revealed that the corrosion within the zinc membrane progressively became deeper, with a maximum depth of approximately 50 μm that was reached after 28 d of immersion. Notably, although the zinc alloy membrane had yet to be fully breached, conspicuous cracks were noted, indicating advanced stages of degradation. Analysis of the elemental distribution within the cross-section indicated that it was primarily composed of zinc, oxygen, phosphorus, calcium, and carbon, with trace amounts of iron and magnesium also present.

The weight loss and corrosion rates are shown in Fig. 2F and G, respectively. After 3 d of immersion, the Zn-0.3Fe-0.05Mg membrane rapidly corroded (0.26 ± 0.038 mm/year) with a weight loss of 7.06% ± 1.05%. The corrosion rate gradually decreased and stabilized, and the Zn-0.3Fe-0.05Mg membrane showed a degradation behavior similar to that of the pure Zn membrane. After 28 d of immersion, the corrosion rate and weight loss of the membrane were 0.18 ± 0.012 mm/year and 19.44% ± 1.32%, respectively, whereas those of the pure Zn membrane were 0.15 ± 0.003 mm/year and 16.11% ± 0.33%, respectively. Overall, the Zn-0.3Fe-0.05Mg membrane exhibited a slightly higher corrosion rate and more weight loss than the pure Zn membrane.

After immersion, the pH value of the Zn-0.3Fe-0.05Mg membrane was initially greater than that of the control group, but its pH was similar to that of the control group after 1 to 7 d. After 14 to 28 d, the pH in the zinc alloy group increased again slightly. Both groups displayed an overall increase in pH with prolonged immersion (Fig. 2I).

Upon immersion in SBF for 28 d, the concentrations of released Zn, Mg, and Fe ions were determined to be 389.46 ± 39.41, 198.12 ± 21.20, and 4.51 ± 0.55 μM, respectively. Notably, an initial burst release of zinc ions was observed on the first day, which increased the concentration of released zinc ions to 198.28 ± 16.37 μM. This initial phase of zinc ion release, which lasted from days 1 to 7, was characterized by rapid kinetics followed by gradual tapering and subsequent stabilization of the release rate (Fig. 2J).

### In vitro cell experiments

#### Concentration of ions in the extract

As shown in Table, the concentration of zinc ions in the membrane extract was 373.33 ± 34.99 μM, significantly differing from that in the control group (*P* < 0.05). In contrast, while the magnesium ion concentration was greater in the membrane extract than in the complete medium, the difference was not significant. Additionally, the iron ion concentration did not differ significantly between the 2 groups (Table). Furthermore, there was no significant difference in pH between the 2 groups (*P* < 0.05). These results showed that the zinc ion concentration was the main difference between the membrane extract and control groups.

**Table.** pH values and ion concentrations in the extract and complete medium

Sample	pH	Zn (μM)	Mg (μM)	Fe (μM)
Zn-0.3Fe-0.05Mg membrane extract	7.47 ± 0.06	373.33 ± 34.99	742.11 ± 24.11	3.39 ± 0.31
Complete medium	7.45 ± 0.04	5.28 ± 0.09	717.42 ± 6.29	4.17 ± 1.36

#### Biocompatibility assessment

**Fig. 3.**
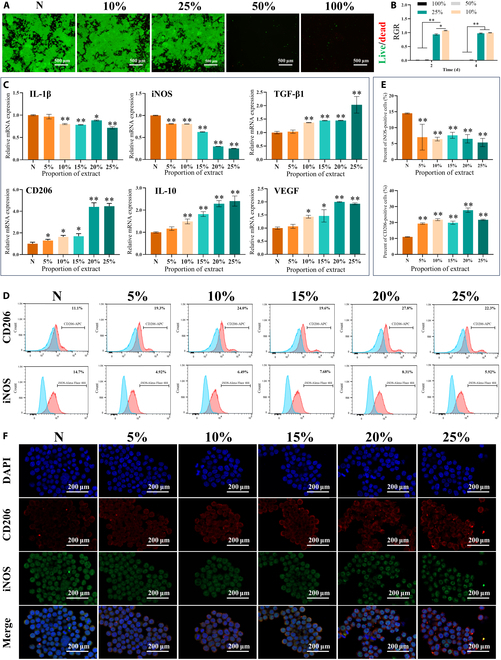
Effects of different concentrations of the Zn-0.3Fe-0.05Mg membrane extract on the proliferation and polarization of Raw264.7 cells. (A) Live/dead staining images of Raw264.7 cells. (B) The relative growth rates (RGRs) of Raw264.7 cells after 2 and 4 d of culture. (C) The expression of inflammation-related genes (IL-1β, IL-10, iNOS, CD206, TGF-β1, and VEGF) in Raw264.7 cells after 3 d of culture, as determined by real-time quantitative polymerase chain reaction (RT-qPCR). (D and E) Protein expression of CD206 and iNOS in Raw264.7 cells after 3 d of culture, as detected by flow cytometry. (F) Representative fluorescence images of CD206 and iNOS staining in Raw264.7 cells after 3 d of culture. The results are shown as mean ± SD. Comparisons were made via one-way ANOVA (*P < 0.05; **P < 0.01). mRNA, messenger RNA.

The cytocompatibility of the Zn-0.3Fe-0.05Mg membrane extracts was evaluated by Cell Counting Kit-8 assays and live/dead staining (Fig. 3A and B). After 2 d of coculture, the higher concentrations (50% and 100%) of the membrane extract demonstrated severe cytotoxicity, with proliferation rates decreasing to less than 1% and marked abnormalities in terms of cell morphology, indicative of obvious damage. In contrast, at lower concentrations (10% and 25%), the membrane extract exhibited remarkable cytocompatibility, as the proliferation rates exceeded 90%, and the cells were healthy and normal in terms of morphology. This trend persisted even after 4 d of coculture, with high concentrations of the membrane extract remaining toxic to the cells, while the cells incubated with low concentrations of the membrane extract exhibited robust proliferation, a substantial increase in the number of macrophages, and the formation of interconnected cell sheets.

#### Immunomodulatory effects on the macrophages

After 3 d of coculture with the Zn-0.3Fe-0.05Mg membrane extract, RT-qPCR analysis revealed notable changes in gene expression in Raw264.7 cells (Fig. 3C). Specifically, in the groups treated with the 10% to 25% membrane extracts, inflammatory genes, such as iNOS and IL-1, were significantly down-regulated, indicating a reduced inflammatory response. Conversely, genes associated with anti-inflammatory and reparative processes, including CD206, IL-10, TGF-β1, and VEGF, were markedly up-regulated.

The expression of the markers iNOS and CD206 was analyzed by FCM to determine the percentages of M1 and M2 macrophages. As shown in Fig. 3D and E, the control group presented a greater percentage of iNOS+ cells than the membrane extract groups, indicating a greater proportion of inflammatory M1 macrophages. Conversely, the percentage of cells expressing CD206, a marker of the anti-inflammatory M2 phenotype, was lower in the control group than in the membrane extract groups.

IF staining revealed the differential expression of CD206 and iNOS in macrophages after exposure to the zinc alloy membrane extract (Fig. 3F). Specifically, CD206, a marker of anti-inflammatory M2 macrophages, was predominantly localized to the surface of the macrophage membrane. In contrast, iNOS, indicative of inflammatory M1 macrophage activation, was found within the macrophages. Notably, in the groups treated with the 10% to 25% membrane extracts, there was a notable increase in CD206 expression and a corresponding decrease in iNOS expression.

#### Osteogenic activity of MC3T3-E1 cells

**Fig. 4.**
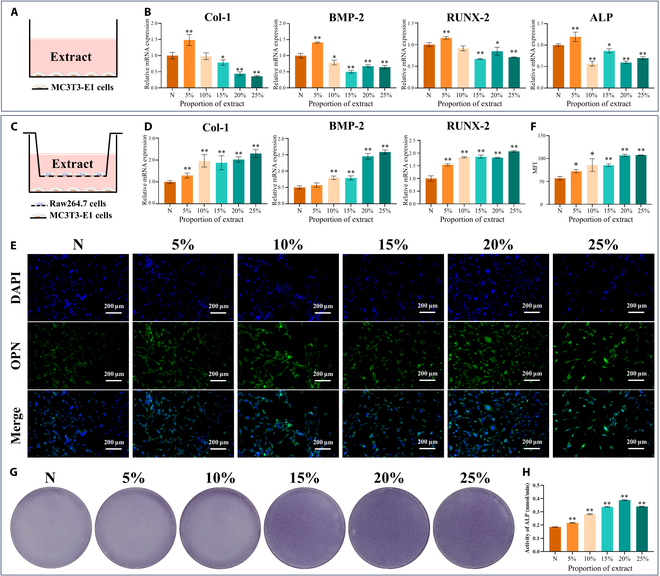
Evaluation of the osteogenic differentiation ability of MC3T3-E1 cells treated with the membrane extract under different cultivation conditions in vitro. (A) Schematic of MC3T3-E1 cells cocultured with the extract. (B) Expression of osteogenesis-related genes (Col-1, BMP-2, RUNX-2, and ALP) in MC3T3-E1 cells after 3 d of coculture with different concentrations of the extracts. (C) Schematic of MC3T3-E1 cells cocultured with the extract in the Raw264.7–MC3T3-E1 coculture system. (D to H) Expression of osteogenesis-related genes and protein in MC3T3-E1 cells cultured with different concentrations of the extracts for 3 d in the Raw264.7–MC3T3-E1 coculture system. (D) Expression of osteogenesis-related genes (Col-1, BMP-2, and RUNX-2). (E and F) Representative fluorescence images of osteopontin (OPN) staining and the mean fluorescence intensity (MFI). (G and H) ALP activity and representative images of ALP staining. The results are shown as mean ± SD. Comparisons were made via one-way ANOVA (*P < 0.05; **P < 0.01).

In the MC3T3-E1 cells incubated with the 5% membrane extract for 3 d, the gene expression of ALP, Col-1, BMP-2, and RUNX-2 increased. In contrast, the 10% to 25% membrane extracts had no significant effect on the expression of these genes (Fig. 4B). These findings suggest that the 10% to 25% membrane extracts had no apparent direct effect on the osteogenic differentiation of MC3T3-E1 cells.

In the 10% to 25% membrane extract Raw264.7–MC3T3-E1 coculture groups, RT-qPCR analysis revealed pronounced up-regulation of the genes BMP-2, Col-1, and RUNX2 in MC3T3-E1 cells after 3 d of coculture (Fig. 4D). Furthermore, IF revealed a significant increase in OPN protein expression in the MC3T3-E1 cells in the 10% to 25% zinc alloy groups after 3 d of coculture with Raw264.7 cells (Fig. 4E and F). These genes and the protein OPN are pivotal for bone formation and regeneration, which suggests that the membrane extracts stimulated osteogenic differentiation.

Additionally, ALP staining and quantitative analysis revealed greater ALP expression and activity in the 10% to 25% membrane extract groups than those in the control group after 3 d of coculture with the zinc alloy extract (Fig. 4G and H). ALP is a key enzyme involved in bone mineralization, and its increased expression and activity indicate accelerated osteogenic differentiation due to the zinc alloy extract.

#### Vascularization of HUVECs

**Fig. 5.**
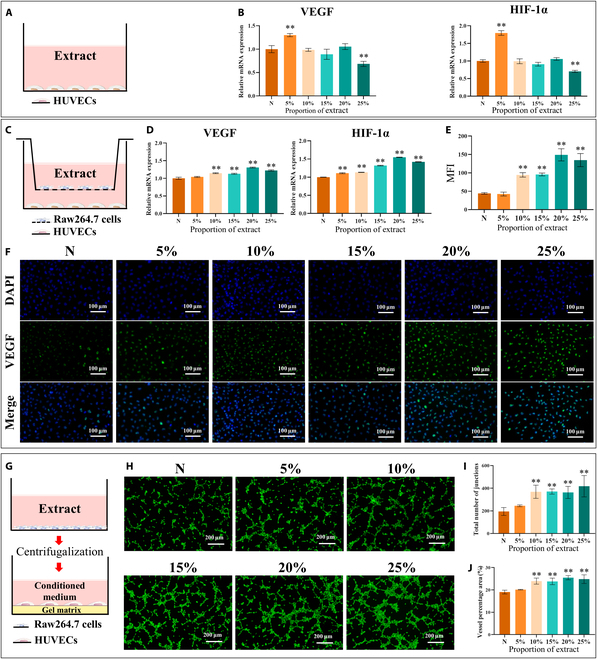
Evaluation of the angiogenic ability of human umbilical vein endothelial cells (HUVECs) treated with the membrane extracts under different cultivation conditions in vitro. (A) Schematic of HUVEC coculturing with the extract. (B) Expression of angiogenesis-related genes (VEGF and HIF-1α) in HUVEC coculturing with different concentrations of the extracts for 3 d. (C) Schematic of HUVECs cocultured with the extract in the Raw264.7–HUVEC coculture system. (D) Expression of angiogenesis-related genes (VEGF and HIF-1α) in HUVECs cultured with different concentrations of extracts for 3 d in the Raw264.7–HUVEC coculture system. (E and F) Representative fluorescence images of VEGF staining and the MFI of HUVECs cultured with different concentrations of extracts for 3 d in the Raw264.7–HUVEC coculture system. (G) Schematic of HUVECs cocultured with the conditioned medium. (H) Representative fluorescence images of tube formation after cultured in conditioned medium. (I and J) Total number of junctions and vessel percentage area in tube formation of HUVECs. The results are shown as mean ± SD. Comparisons were made via one-way ANOVA (*P < 0.05; **P < 0.01).

The 5% membrane extract increased the gene expression of VEGF and HIF-1α in HUVECs after 3 d of incubation, whereas the 10% to 25% membrane extracts had no significant effect on the expression of these genes (Fig. 5A and B). These findings suggest that the 10% to 25% membrane extracts had no obvious direct effect on the vascularization of HUVECs.

Three days after the addition of the zinc alloy extracts to the Raw264.7–MC3T3-E1 coculture system, RT-qPCR analysis revealed markedly increased VEGF and HIF-1α gene expression in HUVECs exposed to the 10% to 25% zinc alloy extracts (Fig. 5C and D). This finding was corroborated by IF staining, which revealed an increase in VEGF protein expression in these groups after 3 d of coculture (Fig. 5E and F). Furthermore, a tube formation assay utilizing CM from zinc alloy extract-treated macrophages revealed the enhanced angiogenic potential of the HUVECs treated with the 10% to 25% membrane extracts, as evidenced by greater total number of junctions and vessel percentage area than those observed in the control group (Fig. 5G and J). These findings indicate that the zinc alloy extract promotes the vascularization of cocultured HUVECs.

### In vivo animal experiments

#### Biocompatibility in vivo

**Fig. 6.**
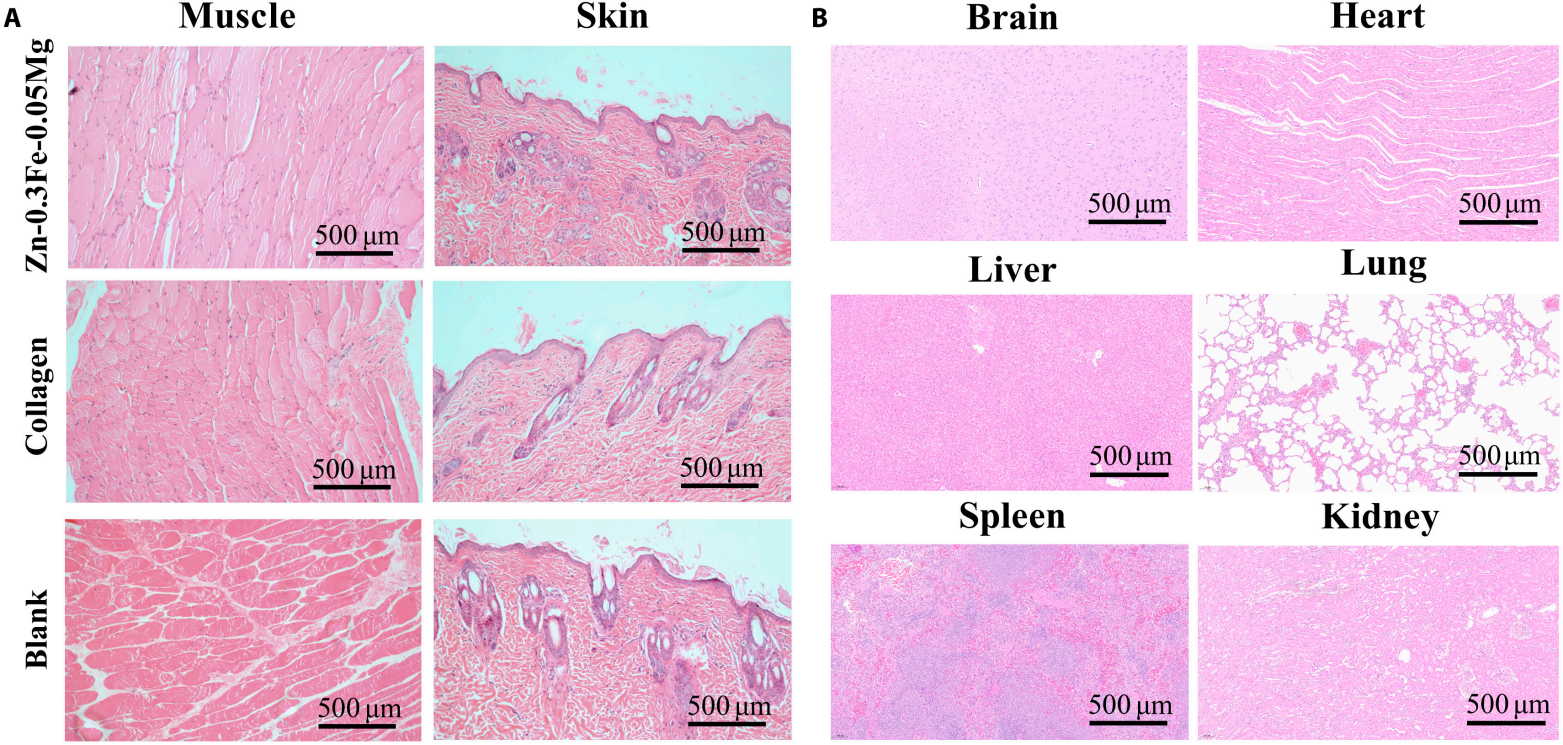
Hematoxylin and eosin (HE)-stained sections of the muscles and skin around the bone defect (A) and major organs (B) from New Zealand rabbits obtained 12 weeks after surgery.

In the New Zealand rabbit skull defect model, HE-stained sections of the soft tissue near the defect area and major organs (brain, lung, liver, spleen, heart, and kidney) 12 weeks after implantation of the Zn-0.3Fe-0.05Mg membranes are shown in Fig. 6. As observed in Fig. 6, the soft tissue near the defect area and major organs (brain, heart, liver, lung, spleen, and kidney) did not exhibit abnormal morphologies at the 12-week mark. There were also no signs of inflammatory cell infiltration, hazardous substance accumulation, or tissue structure disruption.

#### Surface appearance and degradation behavior in vivo

In the New Zealand rabbit skull defect model, surface appearance and degradation behavior in vivo were detected.

**Fig. 7.**
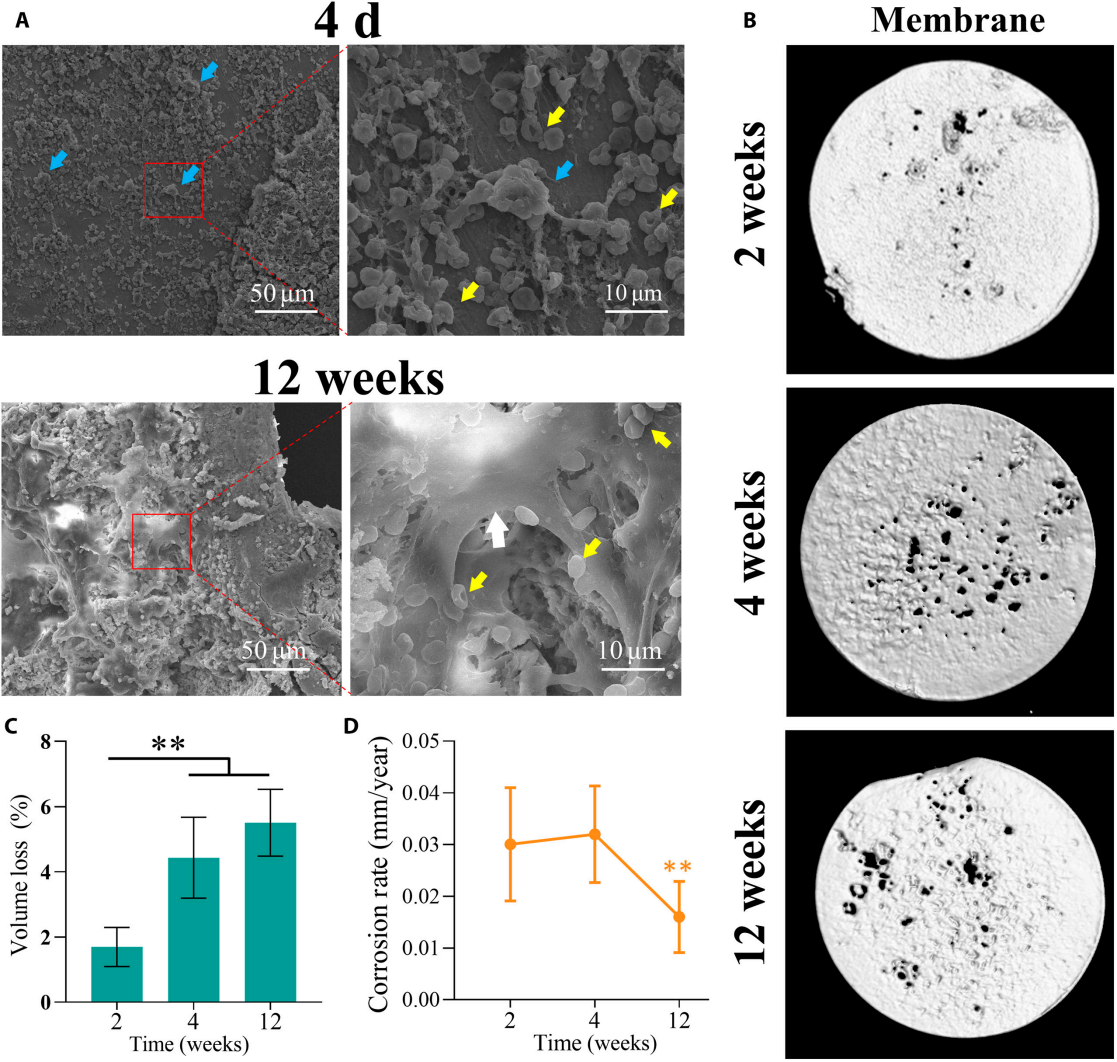
Degradation behavior of the Zn-0.3Fe-0.05Mg membrane in vivo. (A) Surface appearance and cell adhesion of the Zn-0.3Fe-0.05Mg membrane at 4 d and 12 weeks after implantation (blue arrow: inflammatory cells; yellow arrow: red blood cells; white arrow: osteoblast). (B) Representative 3-dimensional (3D) microcomputed tomography (micro-CT) images of the Zn-0.3Fe-0.05Mg membrane taken at 2, 4, and 12 weeks after implantation. (C and D) Volume loss and corrosion rates of the Zn-0.3Fe-0.05Mg membrane. The results are shown as mean ± SD. Comparisons were made via one-way ANOVA (*P < 0.05; **P < 0.01).

Four days after implantation in vivo, SEM revealed that the membrane remained flat without any traces or pit corrosion caused by uneven distribution. There was pronounced adherence of inflammatory cells and red blood cells to the surface of the membrane, which resulted in good cell viability (Fig. 7A). However, at the 12-week mark, many degradation products and new bone tissue were observed on the surface of the membrane. Moreover, rather than inflammatory cells, numerous viable osteoblasts adhered to the membrane surface, indicating that the defect area was in a state of repair (Fig. 7A).

As shown in Fig. 7B, the Zn-0.3Fe-0.05Mg membrane showed noticeable pitting after 2 weeks of implantation, similar to the degradation behavior observed in vitro. After 4 weeks of implantation, the Zn-0.3Fe-0.05Mg membrane had further degraded, and the volume loss rate of the material increased significantly (Fig. 7C, *P < 0.01). However, the membrane remained intact overall and was still capable of acting as a barrier; in addition, its degradation rate did not change significantly (Fig. 7D). Twelve weeks after implantation, spot corrosion of the Zn-0.3Fe-0.05Mg membrane was still the primary type of corrosion observed. The volume loss rate of the material had not increased significantly (Fig. 7C). Moreover, the degradation rate of the material decreased significantly (Fig. 7D), indicating that degradation of the membrane material tended to stall.

#### Immunomodulatory effects on macrophages in vivo

**Fig. 8.**
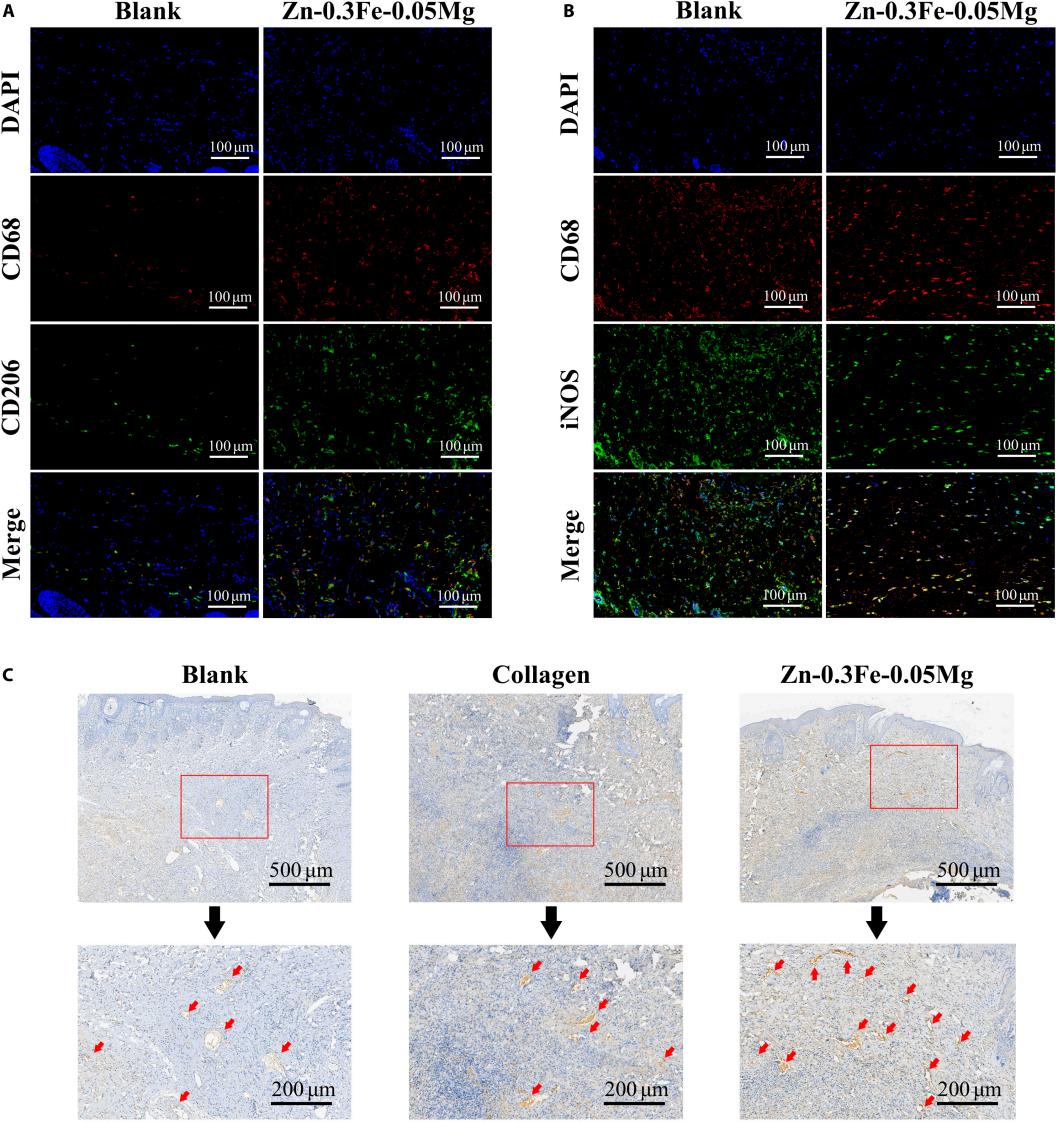
Biological effects of the Zn-0.3Fe-0.05Mg membrane in vivo. (A and B) Representative fluorescence images of CD206+ M2 macrophages and iNOS+ M1 macrophages in the mouse air pouch model. (C) Representative images of CD34+ blood vessels after immunohistochemical staining of the soft tissue around the membrane 1 week after surgery (red arrows: CD34+ blood vessels).

Immunomodulatory effects on macrophages in vivo were detected in the mouse air pouch model. As shown in Fig. 8A and B, the Zn-0.3Fe-0.05Mg membranes presented significantly more CD206+ M2 macrophages than the control group, whereas the number of iNOS+ M1 macrophages was notably lower. These findings suggest that macrophage polarization toward a more anti-inflammatory M2 phenotype occurs in the presence of the Zn-0.3Fe-0.05Mg membrane.

#### Vascularization efficiency in vivo

Vascularization efficiency in vivo was detected in the New Zealand rabbit skull defect model. One week after implantation, the number of CD34+ blood vessels in the Zn-0.3Fe-0.05Mg membrane group was significantly greater than that in the collagen or blank control groups (Fig. 8C). This enhanced angiogenic response indicates that the Zn-0.3Fe-0.05Mg membrane could stimulate the formation of new blood vessels, potentially creating a more favorable environment for tissue regeneration and healing at the implantation site.

#### Osteogenic efficiency in vivo

Osteogenic efficiency in vivo was detected in the New Zealand rabbit skull defect model.

**Fig. 9.**
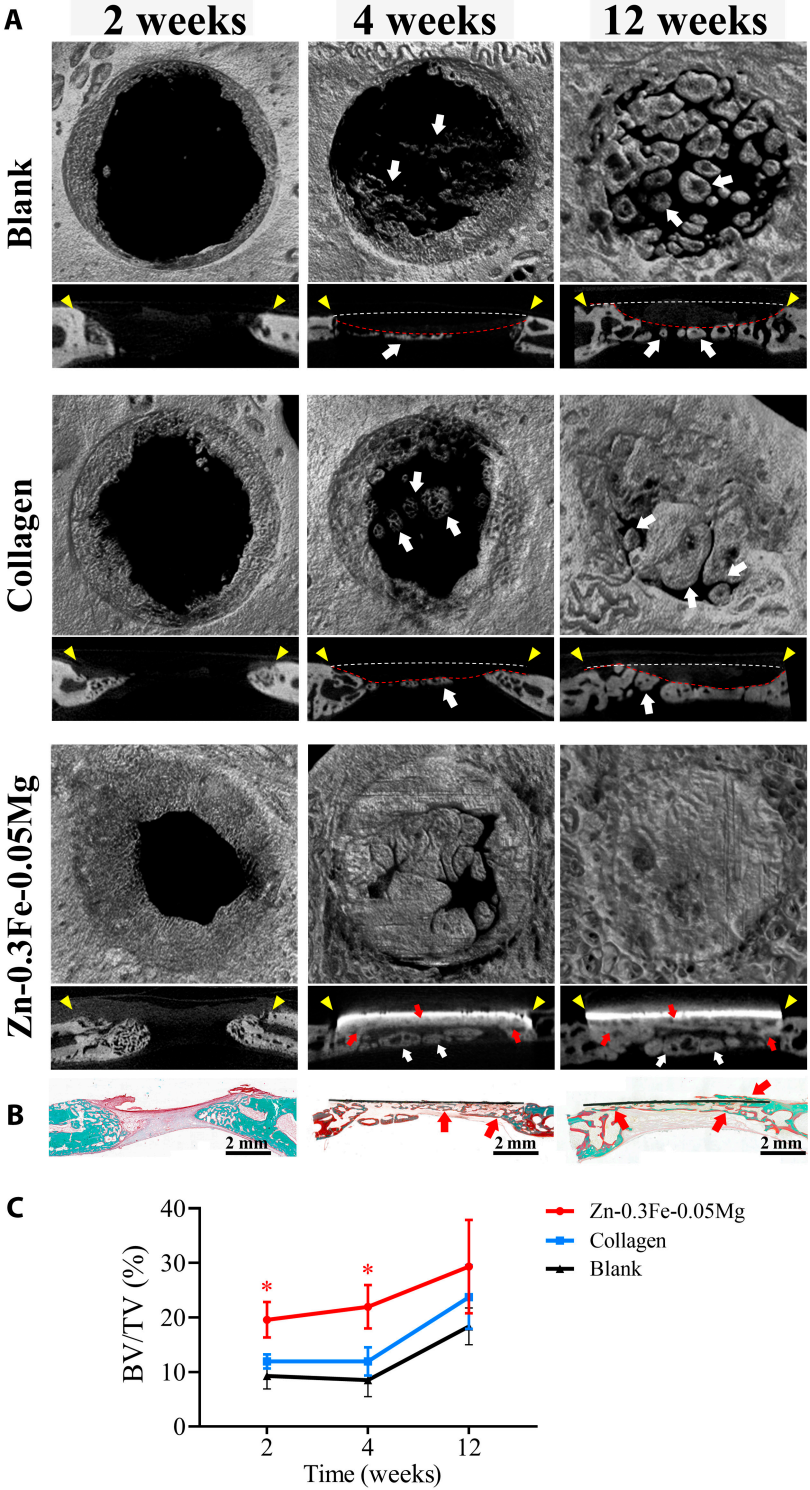
Effects of the Zn-0.3Fe-0.05Mg membrane on guided bone regeneration in vivo. (A) Representative micro-CT images of bone defects at 2, 4, and 12 weeks after surgery. (B) Representative images of Goldner’s staining of the bone defect 2, 4, and 12 weeks after Zn-0.3Fe-0.05Mg membrane implantation (yellow triangle: defect boundary; white arrow: bone island; red arrow: new bone bound to the membrane; white dashed line: outline of the host bone; red dashed line: outline of the new bone). (C) Quantitative analysis of the bone volume/total volume (BV/TV) in the bone defects at 2, 4, and 12 weeks after surgery. (* indicates a significant difference between the Zn-0.3Fe-0.05Mg group and all other groups. The results are shown as mean ± SD. Comparisons were made via one-way ANOVA, *P < 0.05.)

The micro-CT and Goldner’s staining images of the defect area are shown in Fig. 9. Two weeks after implantation, significant new bone formation was observed in the Zn-0.3Fe-0.05Mg group. The BV/TV in the Zn-0.3Fe-0.05Mg group was notably greater than those in the blank and the collagen control groups (Fig. 9C, *P < 0.01). Notably, the Zn-0.3Fe-0.05Mg membrane was not observed in the 2-week group image because the Zn-0.3Fe-0.05Mg membrane had not yet formed a tight bond with the bone tissue at this early stage and had fallen off at the time of detection. However, we can see the Zn-0.3Fe-0.05Mg membrane in Fig. 7B.

Four weeks after implantation, new bone formation increased in all groups. Similar to a previous study by Liang et al. [25], the central part of the critical bone defects in the blank and collagen groups was mainly composed of newly formed island-like bone tissue, which formed independently away from the edge of the defect (white arrow). Therefore, the new bone in these groups lacks sufficient continuity with the host bone. Meanwhile, it can be distinctly observed that the new bone in the blank and collagen groups presents a downward collapsed morphology (red dashed line), compared to the outline of the host bone (white dashed line). In contrast to the island-like osteogenesis in the blank and collagen groups, new bone formed on the meningeal side of the membrane in the Zn-0.3Fe-0.05Mg group and was closely integrated with the membrane (red arrow). The overall morphology of the new bone in the Zn-0.3Fe-0.05Mg group was similar to that of the host bone, and the repair of the critical bone defect in the rabbit skull was essentially complete. The Goldner staining results aligned with the CT data, clearly showing that the red bone matrix and the green mineralized bone progressed along the Zn-0.3Fe-0.05Mg membrane, forming continuous, seamless new bone tissue (Fig. 9B). Quantitative analysis revealed that the BV/TV value in the Zn-0.3Fe-0.05Mg group was significantly greater than those in the blank and collagen control groups (*P < 0.05). However, there was no significant difference between the 2 control groups (Fig. 9C).

Twelve weeks after implantation, the CT coronal view images revealed that although the new bone in the blank and the collagen groups covered the whole defect area, the cross-sectional view images showed that the morphology of the new bone was severely disrupted. Many longitudinal gaps with poor continuity and overall poor bone morphology (red dashed line) were present. In contrast, the new bone in the Zn-0.3Fe-0.05Mg group grew along the membrane and had good continuity with the host bone. In addition, the shape of the new bone was regular with no gaps, and the height and morphology of the host bone were essentially restored. Goldner’s staining revealed that the new mineralized bone grew along the inner and outer surfaces of the Zn-0.3Fe-0.05Mg membrane, completely covered the defect area, and closely integrated with the host bone.

## Discussion

Biodegradable Zn-based materials are considered one of the most promising and marketable GBR barrier membranes because of their biocompatibility, mechanical properties, and degradation behavior [6,8]. However, the insufficient strength and hardness of Zn prevent it from meeting the mechanical requirements of load-bearing orthopedic implants [26,27]. Mg and Fe have been widely used as implant materials because of their good biocompatibility and biodegradability [24]. Notably, Zn-based alloys should avoid the generation of excessive second phases, such as MgZn_2_ and FeZn_13_, as their hardness and brittleness reduce the plasticity of Zn-based alloys, which is not beneficial for GBR [28,29]. Therefore, we prepared Zn-0.3Fe-0.05Mg membranes with Mg and Fe contents of less than 1 wt%.

The Zn-0.3Fe-0.05Mg membrane prepared in this study had a higher YS, hardness, ultimate tensile strength, and elongation at break than the pure zinc membrane, which benefits GBR. Improving the mechanical properties through alloying can provide support to the defect area for GBR applications, leading to better bone regeneration [30]. In studies on zinc-based membranes, restoring the morphology of the defect area is of considerable interest. Figure 9 shows that the Zn-0.3Fe-0.05Mg membrane effectively supported the defect area, resulting in the development of thick bone with a well-formed morphology after 12 weeks in vivo. In contrast, in both the blank and the collagen groups, varying degrees of collapse were observed, leading to the formation of limited quantities of new bone with poor morphology (Fig. 9). In the present study, surgical site collapse also occurred in the pure zinc group [6]. Therefore, the Zn-0.3Fe-0.05Mg membrane we developed significantly improved the mechanical properties compared with those of the pure Zn membrane, as confirmed both in vitro and in vivo.

In addition to mechanical strength, degradability is an important property of GBR materials. The in vitro and in vivo experiments demonstrated that the Zn-0.3Fe-0.05Mg membrane degrades slowly and uniformly under physiological conditions. It is notable that the degradation rates of the Zn-0.3Fe-0.05Mg membrane in vitro and in vivo in this research demonstrated considerable discrepancies. Similar variances have also been witnessed in previous studies [8]. Compared with the corrosion spots and pits on the Zn-0.3Fe-0.05Mg membrane surface in vitro immersion (Fig. 2H), the membrane in the in vivo environment is covered with organic substances such as cells and proteins (Fig. 7A). Furthermore, the dynamic circulation of the in vivo environment is also different from the static immersion experiment in vitro. These reasons affect the contact and reaction between materials and body fluids, causing differences in the degradation rates in vivo and in vitro.

Bone regeneration typically completes 3 to 6 months postsurgery, depending on the size of the bone defect area [6]. Considering the balance between mechanical properties and degradation rate, complete degradation of the membrane should occur after bone regeneration is finished. Additionally, the degradation rate of the membrane affects the concentration of local degradation products, thereby producing different biological effects. Therefore, aspects such as bone regeneration rate, maintenance of mechanical properties, and biological effects are the key factors for evaluating whether the degradation characteristics of Zn-0.3Fe-0.05Mg are conducive to bone regeneration.

At 12 weeks postsurgery, the Zn-0.3Fe-0.05Mg group exhibited favorable bone regeneration efficacy (Fig. 9). At the same time, the Zn-0.3Fe-0.05Mg membrane degraded slowly and remained largely intact (Fig. 7). These results indicate that during bone regeneration, it slowly degrades while maintaining favorable barrier and support functions to promote bone regeneration. In vitro experiments indicated that an appropriate zinc ion concentration promotes bone regeneration. In contrast, excessively high zinc ion concentration exhibits cytotoxicity (Fig. 3). Meanwhile, Zn-0.3Fe-0.05Mg can regulate macrophage polarization toward the M2 phenotype and facilitate early vascularized bone regeneration in the in vivo environment (Figs. 8 and 9). Thus, in vitro and in vivo results confirmed that the rate at which it degrades and releases zinc ions in vivo is favorable for bone regeneration. Unfortunately, the degradation endpoint of the membrane was not observed. In future studies, its longer-term degradation is still needed to investigate further to facilitate clinical application.

Research has indicated that high concentrations of zinc ions are cytotoxic [22,31,32]. In this study, extracts with a concentration less than 25% did not exhibit cytotoxicity, as the zinc ion concentration was less than 93.33 ± 8.75 μM. Additionally, no pathological changes were observed in the soft tissues of the defect area or major organs in the in vivo experiments. Thus, the biocompatibility data of this study are consistent with existing research [22,31–35]. The inconsistency between the in vitro cell experimental results and the animal in vivo data may be because in vitro extraction does not fully reflect the actual in vivo conditions for Zn alloys. In vivo, the degradation products released by degradable metal materials can be quickly removed from the surrounding bodily fluids and excreted without local accumulation, so the concentrations of the degradation products around the implanted material are much lower than that in the in vitro extract [36].

Macrophages can regulate the inflammatory response, which plays a vital role in early bone repair. Promoting M2 macrophage polarization with biomaterials is a major strategy to facilitate early osteogenesis in the defect area [37]. In this study, the extracts promoted M2 macrophage polarization when the zinc ion concentration in the solution ranged from 37.33 ± 3.50 to 93.33 ± 8.75 μM. This regulatory effect may be mediated through multiple pathways. Zinc ions inhibit inflammatory responses by down-regulating the activity of IκB kinase [38]. Additionally, zinc ions can also promote M2 polarization by activating the PI3K/Akt/mTOR pathway [39]. Cui et al. [40] found that ZnMn-based layered double hydroxides promote the expression of metal regulatory proteins to elicit robust M1-like macrophage inhibition.

Furthermore, the regulatory effects of zinc ions on osteogenic differentiation and angiogenesis are concentration dependent. Previous studies revealed that zinc ions at concentrations of 15 and 25 μM increase ALP activity and stimulate the osteogenic differentiation of MC3T3-E1 cells [41]. Additionally, low concentrations of Zn^2+^ promote the osteogenic differentiation of human maxillary sinus membrane stem cells, whereas high concentrations of Zn^2+^ have the opposite inhibitory effect [42]. In addition, Tan et al. [43] reported that 22.5 μM zinc ions up-regulated the genes VEGF and HIF-1α in HUVECs and increased the tube formation ability of the cells. These results are similar to our study’s, which showed that zinc ions at a lower concentration (18.67 ± 1.75 μM) promoted osteogenic differentiation and vascularization.

The animal experiment results of this study demonstrated that the Zn-0.3Fe-0.05Mg membrane significantly promoted bone regeneration in the early stages (2 to 4 weeks). Extensive new bone formation in the early stages may not be due to the action of a single type of cell but rather the result of multiple cell types interacting and collectively promoting bone regeneration. In bone tissue engineering, the interactions among macrophages, osteoblasts, and vascular endothelial cells are of great interest. Li et al. [44] promoted M2 macrophage polarization by sequentially assembling the cytokine IL-4 and RGD peptide onto TiO_2_ nanotubes, thereby enhancing the osteogenic differentiation associated with BMP/Smad/RUNX2 signaling. Some researchers have also reported that 2-dimensional Ti_3_C_2_T*_x_* (MXene) can promote macrophage polarization from the M1 to the M2 phenotype, thereby promoting angiogenesis [45]. The primary effects of macrophages are typically thought to occur in the early stages of injury [46]. This suggests that the interactions among macrophages, osteoblasts, and vascular endothelial cells in this study are key to the ability of the Zn-0.3Fe-0.05Mg membrane to promote early vascularized bone regeneration in vivo.

CM is commonly used to experimentally study the interactions among macrophages, osteoblasts, and vascular endothelial cells. However, CM may exhibit changes in the desired cell-secreted factors and other medium components, such as glucose and serum proteins [47]. In previous studies on zinc-based materials [48], this shortcoming has been particularly critical and yet often overlooked, as the concentration of zinc ions can also change due to nutritional supplementation or the cumulative effects of the CM. The biological effects of zinc ions were confirmed in this study to be significantly concentration dependent. Therefore, conclusions drawn from cocultures using CM may not accurately reflect in vivo conditions. To address this challenge, this study employed transwell chambers for coculture. This approach not only allows the cells to be continuously exposed to secreted factors but also avoids the issue of the changes in ion concentration associated with CM, thereby better simulating in vivo conditions.

Therefore, to further evaluate the effects of the extract on osteogenic differentiation and vascularization resulting from macrophage polarization, we constructed coculture systems of Raw264.7 cells with both MC3T3-E1 cells and HUVECs. The results revealed that the osteogenic differentiation of MC3T3-E1 cells and the vascularization of HUVECs in the coculture systems were effectively promoted when the zinc ion concentration in the extract ranged from 37.33 ± 3.50 to 93.33 ± 8.75 μM. These results indicated that when the zinc ion concentration in the extract was in this range, the macrophages were polarized to the M2 type and secreted pro-osteogenic and pro-angiogenic factors, which further promoted the osteogenic differentiation of osteoblasts and the vascularization of vascular endothelial cells.

To verify their role in inducing macrophage polarization in vivo, Zn-0.3Fe-0.05Mg membranes were implanted into air pouches on the backs of mice. IF showed that, compared with that in the blank control group, the number of M2 macrophages in the Zn-0.3Fe-0.05Mg group was significantly greater, whereas the number of M1 macrophages was significantly lower. Thus, the Zn-0.3Fe-0.05Mg membrane promoted M2 macrophage polarization in vivo.

Vascularization is necessary for bone defect repair [49]. Neovascularization provides the essential oxygen and nutrients for bone tissue. It serves as an important source of mesenchymal stem cells, which play a key role in repairing bone defects [50]. We used CD34 as a marker to detect tissue neovascularization by immunohistochemistry. The results revealed that there were significantly more CD34+ blood vessels in the subcutaneous tissue of the implanted area in the Zn-0.3Fe-0.05Mg group than in the collagen and the blank control groups, suggesting that the Zn-0.3Fe-0.05Mg membrane promoted the formation of new blood vessels in the bone defect area.

Finally, micro-CT and Goldner’s staining were used to fully evaluate bone defect repair in vivo. Importantly, a large amount of new bone extending toward the center of the defect was observed in the Zn-0.3Fe-0.05Mg group. Quantitative analysis revealed that the increase rate of BV/TV in the Zn-0.3Fe-0.05Mg group at 2 to 4 weeks was significantly higher than that at 4 to 12 weeks, which verified the mechanism by which Zn-0.3Fe-0.05Mg promotes early osteogenesis. With increasing implantation duration, the amount of new bone increased in all groups. Small new bone islands were observed at the bottom of the defects in each group [25]. These islands formed independently, away from the edge of the defect, which may have been an attempt by the organism to repair the critical bone defect. However, many longitudinal gaps were observed between the areas of new bone generated in this way, resulting in new bone with an abnormal shape, and poor continuity with the host bone, which were clearly observed in the blank and collagen groups. Furthermore, the morphology of new bone in these 2 groups were depressed downward and failed to restore the original shape of the bone. This may be due to the poor mechanical properties of the collagen membrane, which was unable to fulfill its spatial maintenance function during the long-term repair period. In contrast, owing to the mechanical stability of the Zn-0.3Fe-0.05Mg membrane, the new bone in the Zn-0.3Fe-0.05Mg group grew along the membrane and showed good continuity, a regular morphology, and no gaps, thereby better restoring the shape of the host bone. Notably, we observed that the degradation rate of the Zn-0.3Fe-0.05Mg membrane slowed with increasing implantation duration, and the degradation endpoint was not reached during the 12-week follow-up period.

In conclusion, the developed biodegradable Zn-0.3Fe-0.05Mg membrane with excellent mechanical properties promoted M2 macrophage polarization, thereby promoting early vascularized bone regeneration. However, the degradation endpoint of the membrane was not observed in this study, and thus, the degradation properties of this membrane still need further improvement. Further studies focusing on the composition, structure, and surface treatment of these membrane materials may be needed.

## Conclusion

In summary, a novel biodegradable Zn-0.3Fe-0.05Mg GBR membrane with excellent mechanical properties was prepared, and its effects on osteoimmunomodulation were systematically studied in the present work. The zinc ion concentrations in the 10% to 25% membrane extracts ranged from 37.33 ± 3.50 to 93.33 ± 8.75 μM, and zinc ions at these concentrations effectively fostered the M2 polarization of Raw264.7 cells, which promoted the osteogenic differentiation of MC3T3-E1 cells and significantly enhanced the angiogenic ability of HUVECs. In vivo, the considerable potential of the Zn-0.3Fe-0.05Mg GBR membrane to modulate osteoimmunology and promote early vascularized bone regeneration was clearly demonstrated. These results underscore the potential of the Zn-0.3Fe-0.05Mg membrane as a promising candidate material for GBR.

## Data Availability

All data generated or analyzed during this study are included in this published article.

## References

[1] Alqahtani AM, Moorehead R, Asencio IO. Guided tissue and bone regeneration membranes: A review of biomaterials and techniques for periodontal treatments. Polymers. 2023;15(16):3355. 37631412 10.3390/polym15163355 PMC10457807

[2] Chu C, Deng J, Sun X, Qu Y, Man Y. Collagen membrane and immune response in guided bone regeneration: Recent progress and perspectives. Tissue Eng Part B Rev. 2017;23(5):421–435. 28372518 10.1089/ten.TEB.2016.0463

[3] Fang J, Liu R, Chen S, Liu Q, Cai H, Lin Y, Chen Z, Chen Z. Tuning the immune reaction to manipulate the cell-mediated degradation of a collagen barrier membrane. Acta Biomater. 2020;109:95–108. 32268238 10.1016/j.actbio.2020.03.038

[4] Zhang M, Lin S, Dai Y, Li Y, Zhang D, Wen C. A biodegradable Zn–Se alloy with potent osteogenicity, antibacterial ability, and antitumor efficacy for bone-implant applications. Chem Eng J. 2024;500: Article 156658.

[5] Wen X, Wang J, Pei X, Zhang X. Zinc-based biomaterials for bone repair and regeneration: Mechanism and applications. J Mater Chem B. 2023;11(48):11405–11425. 38010166 10.1039/d3tb01874a

[6] Guo H, Xia D, Zheng Y, Zhu Y, Liu Y, Zhou Y. A pure zinc membrane with degradability and osteogenesis promotion for guided bone regeneration: In vitro and in vivo studies. Acta Biomater. 2020;106:396–409. 32092431 10.1016/j.actbio.2020.02.024

[7] Lee MK, Lee H, Park C, Kang IG, Kim J, Kim HE, Jung HD, Jang TS. Accelerated biodegradation of iron-based implants via tantalum-implanted surface nanostructures. Bioact Mater. 2022;9:239–250. 34820568 10.1016/j.bioactmat.2021.07.003 PMC8586574

[8] Su Y, Fu J, Lee W, du S, Qin YX, Zheng Y, Wang Y, Zhu D. Improved mechanical, degradation, and biological performances of Zn–Fe alloys as bioresorbable implants. Bioact Mater. 2022;17:334–343. 35386444 10.1016/j.bioactmat.2021.12.030 PMC8965087

[9] Shi ZZ, Gao XX, Chen HT, Liu XF, Li A, Zhang HJ, Wang LN. Enhancement in mechanical and corrosion resistance properties of a biodegradable Zn-Fe alloy through second phase refinement. Mater Sci Eng C. 2020;116: Article 111197. 10.1016/j.msec.2020.111197 32806267

[10] Ji C, Ma A, Jiang J, Song D, Liu H, Guo S. Research status and future prospects of biodegradable Zn-Mg alloys. J Alloys Compd. 2024;993: Article 174669.

[11] Jin H, Zhao S, Guillory R, Bowen PK, Yin Z, Griebel A, Schaffer J, Earley EJ, Goldman J, Drelich JW. Novel high-strength, low-alloys Zn-Mg (< 0.1 wt% Mg) and their arterial biodegradation. Mater Sci Eng C. 2018;84:67–79. 10.1016/j.msec.2017.11.021 PMC5846125 29519445

[12] Wang LQ, Ren YP, Sun SN, Zhao H, Li S, Qin GW. Microstructure, mechanical properties and fracture behavior of as-extruded Zn–Mg binary alloys. Acta Metall Sin. 2017;30(10):931–940.

[13] Fu M, Yang C, Sun G. Recent advances in immunomodulatory hydrogels biomaterials for bone tissue regeneration. Mol Immunol. 2023;163:48–62. 37742359 10.1016/j.molimm.2023.09.010

[14] Zhang D, Dang Y, Deng R, Ma Y, Wang J, Ao J, Wang X. Research progress of macrophages in bone regeneration. J Tissue Eng Regen Med. 2023;2023:1512966. 40226416 10.1155/2023/1512966 PMC11919137

[15] Zhang Y, Böse T, Unger RE, Jansen JA, Kirkpatrick CJ, van den Beucken JJJP. Macrophage type modulates osteogenic differentiation of adipose tissue MSCs. Cell Tissue Res. 2017;369(2):273–286. 28361303 10.1007/s00441-017-2598-8 PMC5552848

[16] Zhao X, Zhou X, Sun H, Shi H, Song Y, Wang Q, Zhang G, Xu D. 3D printed Ti-5Cu alloy accelerates osteogenic differentiation of MC3T3-E1 cells by stimulating the M2 phenotype polarization of macrophages. Front Immunol. 2022;13:1001526. 36275667 10.3389/fimmu.2022.1001526 PMC9585254

[17] Wang Y, Fan Y, Liu H. Macrophage polarization in response to biomaterials for vascularization. Ann Biomed Eng. 2021;49(9):1992–2005. 34282494 10.1007/s10439-021-02832-w

[18] Graney PL, Ben-Shaul S, Landau S, Bajpai A, Singh B, Eager J, Cohen A, Levenberg S, Spiller KL. Macrophages of diverse phenotypes drive vascularization of engineered tissues. Sci Adv. 2020;6(18):eaay6391. 32494664 10.1126/sciadv.aay6391 PMC7195167

[19] Pan X, Ou M, Lu Y, Nie Q, Dai X, Liu O. Immunomodulatory zinc-based materials for tissue regeneration. Biomater Adv. 2023;152: Article 213503. 37331243 10.1016/j.bioadv.2023.213503

[20] Qian Y, Zheng Y, Jin J, Wu X, Xu K, Dai M, Niu Q, Zheng H, He X, Shen J. Immunoregulation in diabetic wound repair with a photoenhanced glycyrrhizic acid hydrogel scaffold. Adv Mater. 2022;34(29):2200521. 10.1002/adma.202200521 35576814

[21] Liu W, Li J, Cheng M, Wang Q, Yeung KWK, Chu PK, Zhang X. Zinc-modified sulfonated polyetheretherketone surface with immunomodulatory function for guiding cell fate and bone regeneration. Adv Sci. 2018;5(10):1800749. 10.1002/advs.201800749 PMC6193167 30356934

[22] Chen K, Zhou G, Li Q, Tang H, Wang S, Li P, Gu X, Fan Y. *In vitro* degradation, biocompatibility and antibacterial properties of pure zinc: Assessing the potential of Zn as a guided bone regeneration membrane. J Mater Chem B. 2021;9(25):5114–5127. 34128016 10.1039/d1tb00596k

[23] Zhang W, Li P, Shen G, Mo X, Zhou C, Alexander D, Rupp F, Geis-Gerstorfer J, Zhang H, Wan G. Appropriately adapted properties of hot-extruded Zn-0.5Cu-xFe alloys aimed for biodegradable guided bone regeneration membrane application. Bioact Mater. 2021;6(4):975–989. 33102940 10.1016/j.bioactmat.2020.09.019 PMC7560602

[24] Shao X, Wang X, Xu F, Dai T, Zhou JG, Liu J, Song K, Tian L, Liu B, Liu Y. *In vivo* biocompatibility and degradability of a Zn–Mg–Fe alloy osteosynthesis system. Bioact Mater. 2022;7:154–166. 34466724 10.1016/j.bioactmat.2021.05.012 PMC8379423

[25] Liang L, Wang S, Zhang X, Yan T, Pan X, Gao Y, Zhang X, Wang Q, Qu L. Multi-site enhancement of osteogenesis: Peptide-functionalized GelMA hydrogels with three-dimensional cultures of human dental pulp stem cells. Regen Biomater. 2024;11:rbae090. 39193556 10.1093/rb/rbae090 PMC11349188

[26] Liu C, Li Y, Ge Q, Liu Z, Qiao A, Mu Y. Mechanical characteristics and in vitro degradation of biodegradable Zn-Al alloy. Mater Lett. 2021;300: Article 130181.

[27] Yang Y, Cheng Y, Yang M, Qian G, Peng S, Qi F, Shuai C. Semicoherent strengthens graphene/zinc scaffolds. Mater Today Nano. 2022;17: Article 100163.

[28] Chu X, Fu Z, Liu Y, Dai Y, Wang J, Song J, Dong Z, Yan Y, Yu K. Mechanical properties, microstructure, degradation behavior, and biocompatibility of Zn-0.5Ti-0.5Fe and Zn-0.5Ti-0.5Mg guided bone regeneration barrier membranes prepared using a powder metallurgy method. ACS Biomater Sci Eng. 2024;10(10):6520–6532. 39360994 10.1021/acsbiomaterials.4c01068

[29] Shi Y, Zhang L, Chen J, Zhang J, Yuan F, Shen L, Chen C, Pei J, Li Z, Tan J, et al. In vitro and in vivo degradation of rapamycin-eluting Mg-Nd-Zn-Zr alloy stents in porcine coronary arteries. Mater Sci Eng C. 2017;80:1–6. 10.1016/j.msec.2017.05.124 28866142

[30] Lee H, Won DS, Park S, Park Y, Kim JW, Han G, Na Y, Kang MH, Kim SB, Kang H, et al. 3D-printed versatile biliary stents with nanoengineered surface for anti-hyperplasia and antibiofilm formation. Bioact Mater. 2024;37:172–190. 38549771 10.1016/j.bioactmat.2024.03.018 PMC10972844

[31] Bao G, Fan Q, Ge D, Wang K, Sun M, Zhang Z, Guo H, Yang H, He B, Zheng Y. *In vitro* and *in vivo* studies to evaluate the feasibility of Zn-0.1Li and Zn-0.8Mg application in the uterine cavity microenvironment compared to pure zinc. Acta Biomater. 2021;123:393–406. 33460794 10.1016/j.actbio.2020.12.048

[32] Liu Y, Fu Z, Chu X, Lu Y, Zhang J, Huang J, Liu Y, Yan Y, Yu K. Fabrication and characterization of a Zn-0.5Fe alloy membrane by powder metallurgy route for guided bone regeneration. Mater Res Express. 2022;9(6): Article 065401.

[33] Li P, Dai J, Li Y, Alexander D, Čapek J, Geis-Gerstorfer J, Wan G, Han J, Yu Z, Li A. Zinc based biodegradable metals for bone repair and regeneration: Bioactivity and molecular mechanisms. Mater Today Bio. 2024;25: Article 100932. 10.1016/j.mtbio.2023.100932 PMC10826336 38298560

[34] Ron T, Leon A, Kafri A, Ashraf A, Na J, Babu A, Banerjee R, Brookbank H, Muddaluri SR, Little KJ, et al. Nerve regeneration with a scaffold incorporating an absorbable zinc-2% iron alloy filament to improve axonal guidance. Pharmaceutics. 2023;15(11):2595. 38004574 10.3390/pharmaceutics15112595 PMC10674795

[35] Yamamoto A, Honma R, Sumita M. Cytotoxicity evaluation of 43 metal salts using murine fibroblasts and osteoblastic cells. J Biomed Mater Res. 1998;39(2):331–340. 9457565 10.1002/(sici)1097-4636(199802)39:2<331::aid-jbm22>3.0.co;2-e

[36] Wang J, Witte F, Xi T, Zheng Y, Yang K, Yang Y, Zhao D, Meng J, Li Y, Li W, et al. Recommendation for modifying current cytotoxicity testing standards for biodegradable magnesium-based materials. Acta Biomater. 2015;21:237–249. 25890098 10.1016/j.actbio.2015.04.011

[37] Chen Z, Xing F, Zhou Y, Yu P, Xu J, Luo R, Zhou C, Xiang Z, Rommens PM, Liu M, et al. Integrated osteoimmunomodulatory strategies based on designing scaffold surface properties in bone regeneration. J Mater Chem B. 2023;11(29):6718–6745. 37350139 10.1039/d3tb00727h

[38] Liu MJ, Bao S, Gálvez-Peralta M, Pyle CJ, Rudawsky AC, Pavlovicz RE, Killilea DW, Li C, Nebert DW, Wewers MD, et al. ZIP8 regulates host defense through zinc-mediated inhibition of NF-κB. Cell Rep. 2013;3(2):386–400. 23403290 10.1016/j.celrep.2013.01.009 PMC3615478

[39] Huang X, Huang D, Zhu T, Yu X, Xu K, Li H, Qu H, Zhou Z, Cheng K, Wen W, et al. Sustained zinc release in cooperation with CaP scaffold promoted bone regeneration via directing stem cell fate and triggering a pro-healing immune stimuli. J Nanobiotechnol. 2021;19:207. 10.1186/s12951-021-00956-8 PMC8274038 34247649

[40] Cui X, Huang C, Huang Y, Zhang Y, Wu J, Wang G, Zhou XZ, Zhang J, Wang L, Cheng L, et al. Amplification of metalloregulatory proteins in macrophages by bioactive ZnMn@SF hydrogels for spinal cord injury repair. ACS Nano. 2024;18(49):33614–33628. 39579147 10.1021/acsnano.4c12236

[41] Seo HJ, Cho YE, Kim T, Shin HI, Kwun IS. Zinc may increase bone formation through stimulating cell proliferation, alkaline phosphatase activity and collagen synthesis in osteoblastic MC3T3-E1 cells. Nutr Res Pract. 2010;4(5):356. 21103080 10.4162/nrp.2010.4.5.356 PMC2981717

[42] Zhang Z, Gong N, Wang Y, Xu L, Zhao S, Liu Y, Tang F. Impact of strontium, magnesium, and zinc ions on the in vitro osteogenesis of maxillary sinus membrane stem cells. Biol Trace Elem Res. 2025;203(4):1922–1933. 39150638 10.1007/s12011-024-04303-4

[43] Tan J, Li S, Sun C, Bao G, Liu M, Jing Z, Fu H, Sun Y, Yang Q, Zheng Y, et al. A dose-dependent spatiotemporal response of angiogenesis elicited by Zn biodegradation during the initial stage of bone regeneration. Adv Healthc Mater. 2024;13(4): Article e2302305. 37843190 10.1002/adhm.202302305

[44] Li M, Wei F, Yin X, Xiao L, Yang L, Su J, Weng J, Feng B, Xiao Y, Zhou Y. Synergistic regulation of osteoimmune microenvironment by IL-4 and RGD to accelerate osteogenesis. Mater Sci Eng C. 2020;109: Article 110508. 10.1016/j.msec.2019.110508 32228925

[45] Li T, Ma J, Wang W, Lei B. Bioactive MXene promoting angiogenesis and skeletal muscle regeneration through regulating M2 polarization and oxidation stress. Adv Healthc Mater. 2023;12(4):2201862. 10.1002/adhm.202201862 36427290

[46] Schlundt C, Fischer H, Bucher CH, Rendenbach C, Duda GN, Schmidt-Bleek K. The multifaceted roles of macrophages in bone regeneration: A story of polarization, activation and time. Acta Biomater. 2021;133:46–57. 33974949 10.1016/j.actbio.2021.04.052

[47] Mestres G, Carter S, Hailer N, Diez-Escudero A. A practical guide for evaluating the osteoimmunomodulatory properties of biomaterials. Acta Biomater. 2021;130:115–137. 34087437 10.1016/j.actbio.2021.05.038

[48] Ji H, Shen G, Liu H, Liu Y, Qian J, Wan GJ, Luo E. Biodegradable Zn-2Cu-0.5Zr alloy promotes the bone repair of senile osteoporotic fractures via the immune-modulation of macrophages. Bioact Mater. 2024;38:422–437. 38770427 10.1016/j.bioactmat.2024.05.003 PMC11103781

[49] Lee H, Han G, Na Y, Kang M, Bang SJ, Kang HS, Jang TS, Park JH, Jang HL, Yang K, et al. 3D-printed tissue-specific nanospike-based adhesive materials for time-regulated synergistic tumor therapy and tissue regeneration in vivo. Adv Funct Mater. 2024;34(48):2406237.

[50] Nambiar J, Jana S, Nandi SK. Strategies for enhancing vascularization of biomaterial-based scaffold in bone regeneration. Chem Rec. 2022;22(6): Article e202200008. 35352873 10.1002/tcr.202200008

