# Supplementary material for: A Biodegradable Zinc Alloy Membrane with Regulation of Macrophage Polarization for Early Vascularized Bone Regeneration
Source: Biomater Res. 2025 Jul 2;29:0223. doi: 10.34133/bmr.0223 (PMC12217930; doi:10.34133/bmr.0223)
Supplement: Supplementary 1 — Table S1 [file bmr.0223.f1.docx]

Supplementary Table1.RT-qPCR primers applied in this study

| Gene | Primer sequence (5′-3′) | | | |  |
| --- | --- | --- | --- | --- | --- |
| CD206 (mouse) | | Forward | | GCACTGGGTTGCATTGGTTT | |
|  |  | Reverse | | TGCAGGGTTGACATGAGACC | |
| IL-10 (mouse) | | Forward | | GAGAAGCATGGCCCAGAAATC | |
|  |  | Reverse | | GAGAAATCGATGACAGCGCC | |
| iNOS (mouse) | | Forward | | CTGCTGGTGGTGACAAGCACATTT | |
|  |  | Reverse | | ATGTCATGAGCAAAGGCGCAGAAC | |
| IL-1β (mouse) | | Forward | | TGGAGAGTGTGGATCCCAAG | |
|  |  | Reverse | | GGTGCTGATGTACCAGTTGG | |
| TGF-β1 (mouse) | | Forward | | CAGTACAGCAAGGTCCTTGC | |
|  |  | Reverse | | ACGTAGTAGACGATGGGCAG | |
| VEGF (mouse) | | Forward | | GTCCCATGAAGTGATCAAGTTC | |
|  |  | Reverse | | TCTGCATGGTGATGTTGCTCTCTG | |
| RUNX2 (mouse) | | Forward | | GACACTGCCACCTCTGACTT | |
|  |  | Reverse | | GATGAAATGCTTGGGAACTG | |
| COL-1 (mouse) | | Forward | | CCTAATGCTGCCTTTTCTGC | |
|  |  | Reverse | | ATGTCCCAGCAGGATTTGAG | |
| BMP-2 (mouse) | | Forward | | AACACCGTGCGCAGCTTCCATC | |
|  |  | Reverse | | CGGAAGATCTGGAGTTCTGCAG | |
| GAPDH (mouse) | | Forward | | TGGTGAAGGTCGGTGTGAAC | |
|  |  | Reverse | | CCATGTAGTTGAGGTCAATGAAGG | |
| HIF-1α (human) | | Forward | | GAACGTCGAAAAGAAAAGTCTCG | |
|  |  | Reverse | | CCTTATCAAGATGCGAACTCACA | |
| VEGF (human) | | Forward | | GAGCCTTGCCTTGCTGCTCTAC | |
|  |  | Reverse | | CACCAGGGTCTCGATTGGATG | |
| GAPDH (human) | | Forward | | CAAGAGCACAAGAGGAAGAGAG | |
|  | Reverse | | CTACATGGCAACTGTGAGGAG | |  |

\
